# Development of a UHPLC-UV/Vis Method for Simultaneously Determining Six Beta-Lactam Antibiotics in Plasma: A Tool for the Clinical Implementation of Therapeutic Monitoring of Beta-Lactams

**DOI:** 10.3390/antibiotics14060613

**Published:** 2025-06-17

**Authors:** Iria Varela-Rey, Marta Martínez-Guitián, Gonzalo Hermelo-Vidal, Enrique Bandín-Vilar, Ignacio Novo-Veleiro, Pablo Manuel Varela-García, Irene Zarra-Ferro, Miguel González-Barcia, Cristina Mondelo-García, Anxo Fernández-Ferreiro

**Affiliations:** 1 FarmaCHUSLab, Health Research Institute of Santiago de Compostela (IDIS), 15706 Santiago de Compostela, Spain; iriavarela13@gmail.com (I.V.-R.); m.martinez.guitian@gmail.com (M.M.-G.); zalohermelo@gmail.com (G.H.-V.); enriquebandinvilar@gmail.com (E.B.-V.); irene.zarra.ferro@sergas.es (I.Z.-F.); miguel.gonzalez.barcia@sergas.es (M.G.-B.); 2 Pharmacy Department, University Clinical Hospital of Santiago de Compostela (SERGAS), 15706 Santiago de Compostela, Spain; 3 Home Hospitalization Unit, University Clinical Hospital of Santiago de Compostela (SERGAS), 15706 Santiago de Compostela, Spain; ignacio.novo.veleiro@sergas.es; 4 Internal Medicine Department, University Clinical Hospital of Santiago de Compostela (SERGAS), 15706 Santiago de Compostela, Spain; pablo.manuel.varela.garcia@sergas.es

**Keywords:** beta-lactam, UHPLC-UV/Vis, therapeutic monitoring, simultaneous quantification

## Abstract

**Background/Introduction:** Beta-lactam antibiotics are among the most frequently prescribed drugs in clinical practice, yet their therapeutic drug monitoring remains underutilized despite high interindividual pharmacokinetic variability, especially in critically ill patients. **Methods:** To address this, we developed and validated an ultra-high-performance liquid chromatography (UHPLC-UV/Vis) method for the simultaneous quantification of six beta-lactams (cefepime, ceftolozane, ceftazidime, meropenem, ampicillin, and ertapenem) in plasma. **Results:** This method uses a single gradient mobile phase and a photodiode array detector, ensuring accurate separation, minimal interference, and robust analyte identification. Validation followed EMA bioanalytical guidelines, demonstrating selectivity, precision, accuracy, and linearity within clinically relevant ranges (1.0–50.0 mg/L). Stability tests showed that the analytes were stable in plasma for up to seven days at 4 °C and one month at −20 °C. Pilot clinical implementation in 35 patients revealed significant interindividual variability, supporting the need for routine beta-lactam monitoring. Approximately 26% of trough concentrations were below the minimal inhibitory concentration, while others exceeded thresholds associated with potential toxicity. **Discussion**: This study represents the first UHPLC-UV/Vis method for the simultaneous determination of these six beta-lactams, overcoming limitations of prior methods that required different mobile phases or excluded clinically relevant antibiotics. The method is universally applicable and easily transferable to routine clinical practice. **Conclusions:** These findings underline the importance of beta-lactam monitoring in optimizing treatment outcomes and combating antibiotic resistance in vulnerable populations. Further studies to assess free drug concentrations are warranted to enhance clinical applicability.

## 1. Introduction

Beta-lactam antibiotics are the largest, most diverse, and most widely used family of antibiotics in clinical practice. These antibiotics consist of a beta-lactam ring bound to other radicals, which gives them activity. The modification of these radicals gives rise to the different groups of beta-lactam antibiotics: penicillins, cephalosporins, carbapenems, monocyclic beta-lactams, and beta-lactamase inhibitors [1].

Therapeutic drug monitoring (TDM) is used to optimize the safety and efficacy of many drugs. The need for TDM is based on different factors, including the lack of reliable dose-concentration relationships, interpatient pharmacokinetic variability, relationships between plasma drug concentrations and efficacy or toxicity, and availability of validated drug assays.

Originally, beta-lactams were not considered strong candidates for TDM because they are generally well-tolerated antibiotics [2]. However, in recent years, their TDM has been recommended mainly because of the enormous interindividual variability and alteration of their pharmacokinetics by several situations, particularly in the earlier phase of critical illness [3,4]. These drugs are hydrophilic molecules with a low volume of distribution (Vd). Critically ill patients frequently present changes in interstitial fluid volumes, hypoalbuminemia, and/or extracorporeal support techniques, which can lead to an increased Vd. In addition, certain pathophysiological factors can influence renal clearance, either enhancing it (trauma, burns, early phase of sepsis, or the use of hemodynamically active drugs) or reducing it (renal failure, muscle wasting, and bedridden patients). These changes in renal clearance can, in turn, affect plasma and extracellular antibiotic concentrations. Even though beta-lactams are time-dependent antibiotics, various infectious diseases require the administration of high-dose antibiotics. In these cases, TDM is important to avoid both possible side effects and suboptimal antibiotic concentrations, leading to the development of antibiotic resistance. In addition, nosocomial pathogens (those that cause hospital-acquired infections in vulnerable patients) exhibit high genetic plasticity, so they easily acquire new antibiotic resistance mechanisms [5,6]. Despite the evidence in favour of beta-lactam TDM, the reality is that its clinical implementation is scarce [7].

A determination method applicable to TDM must be rapid, robust, and sensitive for its implementation in clinical practice. There are several published methods for the quantification of beta-lactam antibiotics in plasma for high-performance liquid chromatography with ultraviolet detection (HPLC-UV/Vis). Most of them use different mobile phases to separate the compounds, so that their clinical implementation is difficult [8,9,10,11,12]. The simultaneous determination of many beta-lactam antibiotics would contribute to the implementation of the method in clinical practice, because different patients receiving different medications can be assessed using a single method. In this context, only two previously published articles have analyzed several beta-lactams in a single process [2,13]. However, these methods did not include new carbapenems, such as ertapenem, or the anti-pseudomonal cephalosporin as ceftolozane. It should be noted that *Pseudomonas aeruginosa* is one of the main causes of infections in critically ill patients, which are associated with high mortality rates [14].

Even though the liquid chromatography–tandem mass spectrometry (LC-MS/MS) has some advantages over HPLC-UV, such as higher sensitivity and specificity, smaller sample volume or shorter sample run times, not all hospitals have this equipment or the technical staff qualified to use it, and the advantages it brings are, in many cases, unnecessary for TDM [15]. In addition, although semi-automated methods are an increasingly present option in TDM, they are not available for these antibiotics [16,17]. Therefore, there is an urgent need to develop methods for drug monitoring that are universal and affordable for most hospitals.

We propose a development, validation, and clinical implementation of the first ultra-high performance liquid chromatography (UHPLC) method, which allows for the simultaneous determination of plasma concentrations of the most frequently prescribed beta-lactams: cefepime (FEP), ceftolozane (TOL), ceftazidime (CAZ), cefotaxime (internal standard, IS), ampicillin (AMP), meropenem (MER), and ertapenem (ETP).

## 2. Results

### 2.1. Chromatography

**Figure 1:**
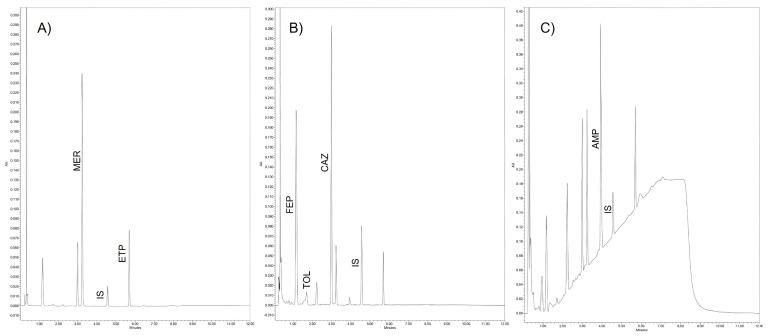
Chromatogram of extracted plasma enriched with working solutions (50 mg/L) at (**A**) 304 nm wavelength, (**B**) 260 nm wavelength, and (**C**) 210 nm wavelength. [meropenem (MER), ertapenem (ETP), cefepime (FEP), ceftolozane (TOL), ceftazidime (CAZ), cefotaxime (internal standard, IS), and ampicillin (AMP)].

**Figure 2:**
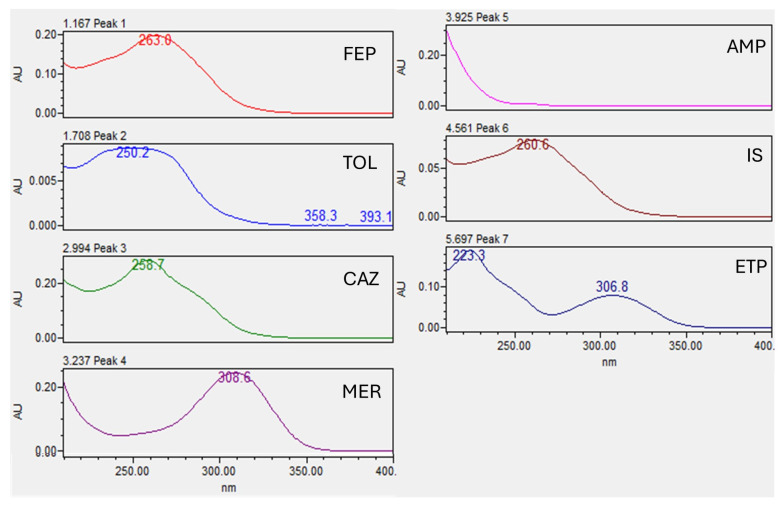
Spectra of different beta-lactam antibiotics [cefepime (FEP), ceftolozane (TOL), ceftazidime (CAZ), cefotaxime (internal standard, IS), ampicillin (AMP), meropenem (MER), and ertapenem (ETP)] obtained with the PDA detector.

The separation of the six compounds was successful. The full analysis takes only 12 min. We have obtained the following retention times with our conditions: 1.2 (FEP), 1.7 (TOL), 3.0 (CAZ), 3.4 (MER), 4.0 (AMP), 4.5 (IS), and 5.8 (ETP) minutes, as illustrated in Figure 1. The gradient used allowed for the correct separation of the analytes and permitted the use of specific wavelengths at 210 (AMP), 260 (FEP, TOL, and CAZ), and 304 (MER and ETP) nm to achieve the highest sensitivity. The photodiode array detector (PDA) incorporated in the analytical equipment and in the processing method allows for the visualization of the spectrum of the analyte in all the analyses, thus providing confidence in the specificity of the method (Figure 2).

### 2.2. Validation of the Method

#### 2.2.1. Selectivity and Carryover

The peaks of the tested antibiotics and the IS are correctly separated in all analyzed tests (see Figure 1). Interference with ceftriaxone, cloxacillin, tazobactam, or avibactam was ruled out. The created library of PDA spectra allows for clearer identification of the six analytes, minimizing the possibility of error; the spectrum for each analyte is shown in Figure 2. After carryover analysis, no contamination was detected in any of the cases, confirming adequate chromatographic conditions.

#### 2.2.2. Calibration Curve and Lower Limit of Quantification (LLOQ)

Calibration lines were adequately described by linear regression. The percentage deviation from nominal was adequate (<15%) for all the points except for the concentration of 1 mg/mL for ETP and FEP and 1 and 2.5 mg/mL for TOL, so these points were excluded from the linearity curve of these analytes. These limit concentration values were also selected as the lower limit of quantification (LLOQ) and evaluated in this context, obtaining appropriate values (<9% of mean relative error, MRE). The linearity details, such as calibration range, equation, coefficient of determination, and precision of LLOQ, are shown in Table 1. The equation parameters and the coefficient of determination are shown as the mean obtained from the five calibration curves, and the %MRE is also shown as the mean of the five replicates analyzed for the LLOQ.

#### 2.2.3. Accuracy and Precision of Quality Controls and Dilution Integrity

The between-day and within-day replicate analysis of quality controls demonstrated a high level of precision and accuracy of the method. In the same case, precision and accuracy measurements for concentrations above the upper limit of quantification (ULOQ) demonstrate the robustness of the method for cases where sample dilution is necessary. Data from both assays are available in Table 2.

#### 2.2.4. Internal Validation

**Figure 3:**
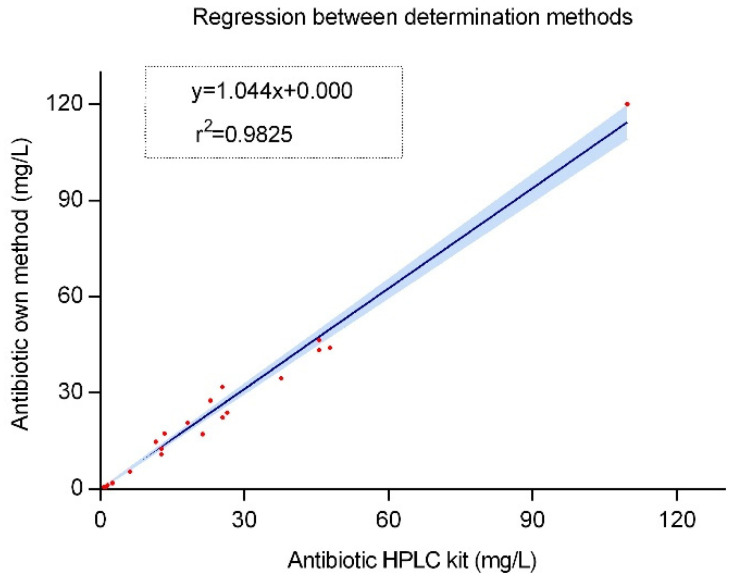
Internal validation. Correlation between two analytical methods: HPLC commercial kit by Chromsystems and our own analytical method developed. The blue area represents the 95% confidence bounds.

A total of 22 patients treated with beta-lactams were included: 4 with AMP, 4 with FEP, 4 with CAZ (2 of them with ceftazidime/avibactam and 2 with only ceftazidime), and 10 with MER. The correlation analysis was performed between the own developed method, and the antibiotic HPLC kit 61,000 marketed by Chromsystems Instruments & Chemicals GmbH (Gräfelfing, Germany) [18] showed very good results. The percentage of the coefficient of variation (CV%) of the concentration obtained with the two methods was less than 20% in all cases, the mean CV% being 10.71 ± 5.9%. The measurements with the greatest deviation (a maximum of 19%) are related to low concentrations, close to the limit of quantification of the analyte. The correlation is clearly linear, with a coefficient of Pearson of r = 0.9912 (see Figure 3). In no case would the differences obtained represent a clinical paradigm shift.

### 2.3. Stability

All antibiotics (working solutions) were stable in water for 6 months, stored at −20 °C, except ETP, and the IS were stable in the same conditions for 9 months (Table 3).

On the other hand, analytes in extracted plasma were stable in the autosampler at 6 °C for 7 days (Figure S2 in Supplemental Material) and at −20 °C for 1 month (Table 4). In both trials, the difference between the concentrations of fresh and frozen or stored antibiotics in the autosampler was less than 20%.

Finally, only cefepime and ampicillin were stable in blood for the first 8 h, both at RT and at 4 °C. The percentage of remaining drug concentration was higher than 88% in the different conditions, as shown in Figure S1 in the supplemental material. For all other antibiotics, there was a decrease in the percentage of remaining drug concentration below 85% in the first 8 h. The antibiotic that showed the lowest stability in blood was meropenem, with 43% degradation at 72 h. In the case of the stability in plasma, all antibiotics were stable for 7 days with a remaining drug percentage above 90%, as shown in Figure S1 in the Supplemental Material.

### 2.4. Clinical Implementation

**Figure 4:**
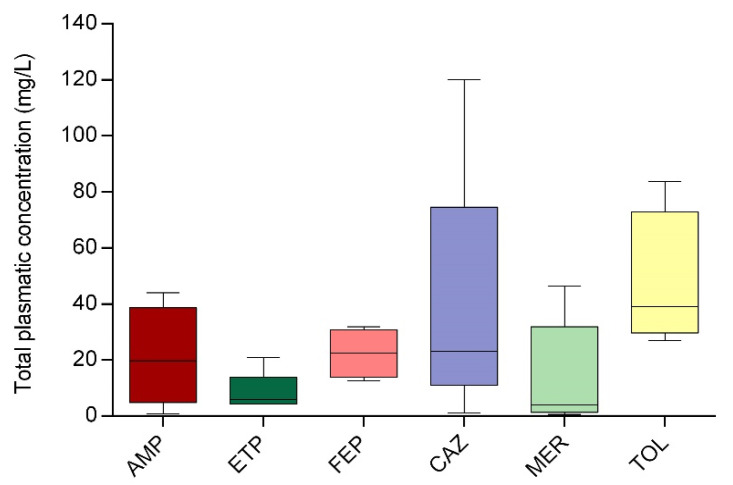
Total plasmatic concentration (mean and SD) of antibiotics in the pilot clinical study.

A total of 35 patients treated with beta-lactams were included: 4 with AMP, 5 with ETP, 4 with FEP, 6 with CAZ (3 of them with ceftazidime/avibactam and 3 with only ceftazidime), 12 with MER, and 4 with TOL. A total of 54.3% were patients admitted to normal hospitalization areas, including nephrology, hematology, infectious diseases, cardiology, pneumonology, urology, and surgery; 34.3% were patients admitted to critical care areas, and 11.4% were patients admitted to hospitalization at home. There were no problems during sample collection or transport to the analysis site, with a maximum time from extraction to freezing of 30 min. All samples could be successfully analyzed, the CV% between the concentrations determined on different days was always less than 15%, the average being 3.1%. Figure 4 shows the mean concentrations obtained for antibiotics, as well as the standard deviation (SD). Only on three occasions there was a concentration below the LLOQ observed, so the result was expressed as less than the LLOQ; in another 3 cases, it was necessary to dilute the sample.

The results obtained show a large interindividual variability (see Figure 4). In addition, analyzing the results from a clinical point of view, we observed that 25.7% of free concentrations were below minimum inhibitory concentration (MIC) (setting the breakpoint at the worst-case scenario). Among the 74.3% that were above MIC, 57.7% were at 1–4 times above MIC, 23.1% were 4–8 times above MIC, and 19.2%, 8 times above MIC, which could be related to potentially toxic concentrations [3,19].

## 3. Discussion

The clinical implementation of the analytical determination of new drugs is hampered by many aspects, such as the lack of adequate infrastructure and personnel for the development of new methods and the difficulty of subsequent transfer to clinical practice [20]. For this reason, it is essential to develop simple, rapid analytical methods that allow the analysis of several analytes at the same time. Liquid chromatography, despite being a complex technique that requires qualified technical personnel, is an analytical determination technique that has been adapted for clinical use, and more pharmacy services have UHPLC-UV, while UHPLC-MS/MS is still a minority, mainly due to its difficulty in acquisition and high level of technical expertise [21].

The work presented here is the development and validation of a chromatographic method for the simultaneous determination of 6 beta-lactam antibiotics in plasma in only 12 min and without mobile phase change. Simultaneous determination facilitates the implementation of the method in clinical practice. To date, only two studies have been published in which beta-lactam antibiotics have been simultaneously by chromatography coupled to an ultraviolet/visible detector [2,13]. The main difference between these methods and the one presented here is that they do not include AMP, ETP, or the anti-pseudomonal cephalosporin TOL. ETP and TOL are both widely prescribed antibiotics for critically ill patients. These patients have great inter-individual variability in achieving plasma concentrations, making them great candidates for TDM.

As for the sample preparation, we performed a protein precipitation with acetonitrile, followed by a lipid removal with chloroform, as well as McWhinney et al. [9]. In order not to affect the matrix effect, dilutions were made in the sample prior to the injection into the chromatographic system. The most diluted antibiotic was TOL, as it is the most affected by the matrix effect. In fact, most of the published studies determine TOL plasma concentrations by mass spectrometry [22,23,24], very few by chromatography coupled to a UV/Vis detector [25] and none by simultaneous determination with other antibiotics.

Mobile phases were a phosphate solution 10 mM (A) and acetonitrile with formic acid 0.1% (B). The acetonitrile gradient allowed elution of the compounds by polarity. The separation of the peaks was good; however, the pH of the mobile phase A was 2.4, as it favours the separation of CAZ and MER. Slight changes in pH modified the retention time of antibiotics [6,8].

The implementation of the PDA detector in the processing method provides a plus in the correct identification and makes it possible to check the absence of interferences that prevent the correct determination of the analyte in a specific patient, an aspect that has already been referred to as a limitation of chromatographic methods [26]. In future steps, it would be appropriate to check the absence of interferences also by UHPLC-MS/MS.

The validation of the analytical method, following the European Medicines Agency (EMA) bioanalytical method validation guide, with the addition of the comparison with the marketed kit, and endorsed by the ISO standard, guarantees an adequate performance of the developed method [27,28]. The LLOQs achieved are similar to others described in the literature, even similar to those described for UHPLC mass tandem determination methods, with higher sensitivity [29,30,31,32,33]. These concentrations are sufficient for clinical implementation, as they allow us to see whether the concentration is below or above (and how many times) the MIC, the current PKPD target for beta-lactams [3,4,5,7,34,35]. A higher sensitivity that would allow detection of concentrations below 1 mg/L is not necessary in this case, so the developed method is optimal [36].

The precision and accuracy values were worse in the between-day analysis than in the within-day analysis, as expected, but they were found to neither exceed 15% for QC samples, thus conforming to the EMA criteria, and were similar to previous publications [29,30,31,32,33]. These results indicate that the proposed method provides acceptable precision and trueness.

The test carried out for the internal validation of the method, comparing it with the values analyzed by the commercial kit, provides great value and security to the development of the method and highlights this work compared to others [29,30,31,32,33] that have not been validated directly against other analytical methods. The limit of variation allowed between different methods is not clear, the correlation test being the most appropriate tool to compare. Linear regression analysis demonstrated a slope of 1.044, indicating a minimal increase in concentration over time. The exceptionally high R-square value of 0.9825 suggests a robust fit of the linear regression model, highlighting the consistency of the method development.

Commercial kits provide security, as they are validated according to International Organization for Standardization (ISO) standards and endorsed by the marketing company; they also simplify the processing of samples and allow for maximum standardization of the analysis, reducing possible analytical errors. However, their use also has negative aspects, such as the lack of knowledge of the reagents used and the limitation in terms of the antibiotics determined. The developed method itself includes more antibiotics, is more versatile, and provides more information due to the knowledge of each of the chromatographic reagents used; the only drawback is the processing time, although it is still manageable if technical staff are available.

The stability study allows the use of the method to be adapted for clinical implementation. The stability tests were performed based on the peak area. Therefore, the 15% variation cannot be set as a limit, as it refers to the nominal concentration. In water stability tests at −20 °C at 6 months, carbapenem antibiotics had a higher difference, and therefore, a reduction in the storage time is recommended for these analytes. Carbapenems also showed a greater deviation in the stability at −20 °C and for one month of the processed samples but were within the accepted limits. This agrees with the literature related to the stability of carbapenems [37,38]. The samples processed and stored in the autosampler were stable for at least 7 days. Other works measured stability in the autosampler for 12–24 h [8,9]. Therefore, we have scope for long sequences.

On the other hand, only FEP and AMP were stable in blood during the first 8 h after blood collection [39], so it is recommended to centrifuge the sample to obtain the plasma as soon as possible. Plasma samples were stable both at 4 °C and frozen at −20 °C for at least 7 days. This is in line with already published stability data [37].

The pilot study demonstrates the validity of the method for clinical implementation. It is important to highlight the variety of the types of patients included in the study, including both critically ill patients and patients in hospitals at home, which reinforces the versatility of the method developed. Most of the antibiotic concentrations were found to be within the range of the calibration line, which facilitates their interpretation. A high inter-individual variability between patients was observed, which is consistent with the high variability in beta-lactam plasma concentration previously described in the literature, and one of the main reasons why TDM is currently recommended [3,40,41,42,43]. Furthermore, despite being a pilot study, the analysis of the results shows the variability in terms of the concentration observed, highlighting that approximately 26% were below MIC and would require a dose increase and 14% of the total were very high (8–10 times above MIC) and would require a dose decrease. The data therefore show that approximately 40% of the patients included would require a change in dosage. This data is consistent with previously published literature, although it is true that most of the cases refer to critically ill patients, which is not the case in this study [40]. This discordance may be because we have taken the highest possible breakpoint, which is the most common scenario in critically ill patients.

This work has some limitations. Firstly, the use of commercial vials of cefepime, ertapenem, and Zerbaxa^®^ (ceftolozane/tazobactam) for method validation was justified due to the limited availability. To reduce possible interferences, the standards were used once to compare them with the commercial ones; due to the similarity and the absence of excipients that would generate interferences, it was decided to use the commercial ones for the validation of the method. Another limitation is the large sample volume required (minimum 500 µL of plasma) for the analysis and processing of the samples, which involves numerous steps requiring time and skilled personnel. On the other hand, due to the unavailability of the analysis of ceftolozane and ertapenem by commercial HPLC/UHPLC kits, in-house validation has not been performed for these two analytes.

Finally, it is important to emphasize that the method has been validated for the determination of total antibiotic concentration, not free antibiotic concentration. The analysis of the clinical results of the pilot study has been performed by estimating the free concentration according to literature data and using the worst-case scenario according to the European Committee on Antimicrobial Susceptibility Testing (EUCAST) breakpoint, which could bias the interpretation of the results. However, the determination of total concentration is still the standard practice in most hospitals, mainly due to its lower cost and the moderate protein binding of beta-lactams [44,45]. In further studies, it would be interesting to optimize the method for the determination of free drug.

## 4. Materials and Methods

### 4.1. Reactives

Ampicillin, cefotaxime, ceftazidime, and meropenem were purchased as Pharmacopeia reference standards (USP) from Sigma-Aldrich (Darmstadt, Germany). Ertapenem, cefepime, and ceftolozane were obtained as drugs commercial drug formulations for injection (Ertapenem^®^ from Aurovitas, Madrid, Spain; Cefepima^®^ from Laboratorio Reig Jofre, Sant Joan Despí, Spain, and Zerbaxa^®^ from Merck, Darmstadt, Germany). Prior to the use of the commercial vials, the purity was experimentally compared with reference standards.

Acetonitrile > 99.9% purity grade and formic acid were purchased from Thermo-Fisher (Waltham, MA, USA); sodium dihydrogen phosphate anhydrous was obtained from Merck (Darmstadt, Germany); water, orthophosphoric acid 85%, and chloroform were purchased from VWR Chemicals (Leicestershire, UK).

### 4.2. Preparation of Stock Solutions and Working Solutions

Stock solution concentrations were 3000 mg/L for all antibiotics, except for IS, which was 500 mg/L. These solutions were prepared in water, pooled, and stored at −20 °C. Working solutions, at the concentrations of 500, 300, 200, 100, 75, 50, 25, and 10 mg/L, were obtained through dilutions from the pool stock solution, and they were also stored at −20 °C.

### 4.3. Sample Preparation

**Figure 5:**
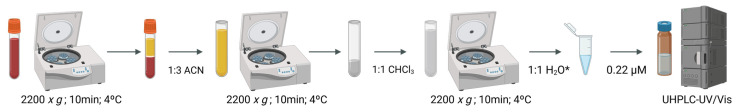
Sample preparation * Dilution 1:3 for TOL.

Calibration standards were validated in the range of 1.0–50.0 mg/L. Calibration standards were freshly prepared by dilution of the respective stock or working solution and IS in human plasma free of analyte prior to extraction. The process is showed in Figure 5. Blood from patients has been collected in EDTA tubes prior to infusion of the beta-lactam antibiotic (trough concentration or Cmin) and centrifuged at 2200× *g* for 10 min at 4 °C to obtain plasma. Both calibration standard and plasma samples were prepared as follows: 25 µL of IS was added to 500 µL of the sample. Protein precipitation was performed with 1500 µL of acetonitrile, vortexed, and centrifugated under the same conditions. Supernatants were collected and treated with chloroform in a proportion of 1:1 *v*/*v* for lipid extraction, vortexed, and centrifugated again. Supernatants were collected and diluted 1:3 *v*/*v* for TOL and 1:1 for the rest of the antibiotics. Finally, 500 µL of this mixture was transferred to vials, filtered through a 0.22 µm PTFE filter (STF020015H/L, CHMLAB Group, Barcelona, Spain), and analyzed by ultrahigh-performance liquid chromatography (UHPLC) with UV-Vis.

Quality control (QC) samples were prepared by spiking known volumes of beta-lactam antibiotics from working solutions to drug-free human plasma to obtain three concentrations at low (5.0 mg/L), medium (20.0 mg/L), and high levels (50.0 mg/L). Lower and upper limits of quantification were also prepared by diluting the working solutions in plasma.

### 4.4. Chromatography System

The UHPLC system consisted of a Waters Acquity H-Class Plus UPLC (Waters Corporation, Milford, MA, USA) equipped with a PDA detector and a reverse-phase Luna^®^Omega C18 column (1.6 µm, 50 × 2.1 mm, 100 Å; Phenomenex Kinetex, Torrance, CA, USA). Mobile phases consisted of 10 mM sodium dihydrogen phosphate solution acidified with orthophosphoric acid to pH 2.4 (Phase A) and acetonitrile with 0,1% formic acid (Phase B). For this purpose, 10 μL of each sample at 30 °C was injected, and the flow rate was set to 0.5 mL/min with controlled room temperature (RT). A programmed mobile-phase gradient was used: 0.5 min. 96% A–4% B; 6 min. 82% A–18% B; 7.50 min. 82%A–18% B; 7.60 min. 96% A–4% B; 12 min. 96% A–4% B. The same IS was used for all antibiotics, but at different wavelengths.

### 4.5. Validation of the Method

The correct validation of the method is critical to ensure that the results are suitable for their intended purpose; with this aim, it was conducted with reference to the EMA guideline on bioanalytical method validation [27].

#### 4.5.1. Selectivity

To investigate interferences, ten blank plasma samples from the plasma of different real patients were prepared and analyzed individually.

Specificity of the assay was demonstrated by confirming the absence of chromatographically interfering peaks from co-drugs.

In addition, we intentionally tested for possible interference from ceftriaxone, cloxacillin, tazobactam, and avibactam, drugs that might be quite likely to be present in beta-lactam TDM candidate patients. As recommended, the absence of interfering components was accepted when the blank responses were lower than 20% of the LLOQ for the compounds and 5% for the corresponding IS. Finally, to improve safety in selectivity, a library of PDA spectra for each of the antibiotics was created and compared for each of the measurements.

#### 4.5.2. Carryover

Carryover was assessed by measuring the signal of five blank samples analyzed directly after the high-concentration standard. According to recommendations, the signal should not exceed 20% of the LLOQ and 5% of the IS.

#### 4.5.3. Calibration Curve and Lower Limit of Quantification (LLOQ)

Linearity for each antibiotic was tested by extracting plasma standards spiked at nominal concentrations of 1.0, 2.5, 5.0, 7.5, 10.0, 20.0, 30.0, and 50.0 mg/L prepared from aqueous standards. The calibration line was generated by least squares linear regression of the peak area ratio of the analyte/IS against nominal concentration by Empower software version 3.8.0.1 (Waters Corporation, Milford, MA, USA). The percentage deviation from the nominal concentration was calculated later at each standard concentration, with ≤15% (≤20% at LLOQ) as the acceptance criterion for inclusion in the calibration curve. Five calibration curves were performed for each antibiotic on different days. The mean, standard deviation (SD), and coefficient of variation (%CV) of concentration of each point, slope, and coefficient of determination were calculated, as well as the mean relative error percentage (%MRE) of concentration of each point. The lower limit of quantification (LLOQ) was validated by five replicate analyses of plasma spiked at 1.0, 2.5, or 5.0 mg/L, depending on the analyte.

#### 4.5.4. Precision and Accuracy

Within-run and between-run precision and accuracy of the assay were assessed by replicate analysis (N = 10) of the low (5 mg/L), medium (20 mg/L), and high (50 mg/L) plasma quality controls.

Concentrations were determined from the calibration curves, and the precision (%CV) and accuracy (%MRE) were calculated at each level. A percentage of 15% was set as the allowable deviation limit for both accuracy and precision.

#### 4.5.5. Dilution Integrity

Dilution integrity was demonstrated for each analyte by spiking the matrix with concentrations of analytes above the upper limit of quantification (ULOQ), 100 mg/L (ULOQ I) and 150 mg/L (ULOQ II). Within-run and between-run precision and accuracy were measured in ten replicates.

#### 4.5.6. Internal Validation

In order to test the validity of the developed method, samples of real patients from different clinical areas of the University Hospital of Santiago de Compostela were analyzed and the concentration was measured in parallel with this method and with the antibiotic HPLC kit 61,000 marketed by Chromsystems Instruments & Chemicals GmbH (Gräfelfing, Alemania) and certified according to the ISO 13485 standard [18]. The correlation was analyzed by linear regression. Since there are no commercially available kits that measure ceftolozane or ertapenem, these two analytes could not be tested in this step.

### 4.6. Stability Assay

The stability of the working solutions and IS was evaluated by comparing a solution that had been stored at −20 °C for 6 or 9 months with a freshly prepared one. Concentrations used were 5 and 50 mg/L. Three replicates have been made. Additionally, the stability of the IS and analytes in extracted plasma was tested by placing processed samples in glass vials within the autosampler at 6 °C for 7 days and storing at −20 °C for 1 month. The areas under the chromatographic peaks (peak area) were compared, along with the areas normalized to the IS. Three replicates have been made.

On the other hand, the stability of beta-lactam antibiotics in both whole blood and plasma has been assessed, too. Blood was collected in EDTA tubes and enriched with working solutions at the following concentrations: 5, 20, and 50 mg/L. Aliquots were taken at 0, 8, 12, 24, 48, 72 h. Stability was tested at 4 °C and at RT. In the other case, human plasma free of analyte was enriched with working solutions at the same concentrations. In this case, aliquots were taken at 0, 12, 24, 48, 72 h, 4, 5, 6, and 7 days. Stability was tested at 4 °C and at −20 °C. The samples were processed as explained above and analyzed by UPLC/UV-Vis. Three replicates were made.

### 4.7. Clinical Implementation

With the aim of transferring the developed method to clinical practice and implementing therapeutic monitoring of beta-lactams, a pilot study was carried out in patients admitted to different clinical areas of the hospital (normal hospitalization area, critical care area, and home hospitalization). The study was approved by the clinical research Ethics Committee with registered number 2020/486. In order to evaluate the analytical capacity of the method and the feasibility and the relevance of its clinical implementation, two blood samples were collected (EDTA anticoagulated tube) prior to infusion of the beta-lactam antibiotic (trough concentration or Cmin). The samples were immediately centrifuged, and the plasma was frozen at −80 °C until analysis. Each of the samples collected for each patient was analyzed on different days; the results are shown as the mean ± standard deviation (SD) and CV%. Subsequently, the free drug concentration (fC_min_) was determined (based on literature data on plasma protein binding [46]), and the number of times the concentration was found to be above the MIC (using the worst-case scenario MIC value and using the breakpoint data recorded in EUCAST in December 2024 [47]).

## 5. Conclusions

The determination of beta-lactam antibiotics in plasma is essential for the monitoring of these antibiotics. Despite the advantages of UHPLC-MS/MS over UPLC-UV/Vis, the development and validation of UPLC-UV/Vis’s determination methods are very important because not all hospitals have a UHPLC-MS/MS system available. The proposed method quantifies six beta-lactam antibiotics (FEP, TOL, CAZ, MER, ETP, and AMP) simultaneously without mobile phase change, which facilitates their implementation in clinical practice. Moreover, it is the first method in which TOL, ETP, and AMP are quantified together with other antibiotics by UPLC-UV/Vis. The method employs a single-gradient mobile phase coupled with a PDA detector, enabling precise separation, reduced interference, and reliable analyte identification. Validation was conducted in accordance with EMA bioanalytical guidelines, confirming its selectivity, precision, accuracy, and linearity across clinically relevant concentrations (1.0–50.0 mg/L). Furthermore, the validity of the method was confirmed using commercial kits. A pilot study of 35 patients confirmed the need for TDM in beta-lactam antibiotics due to their high inter-individual variability and the validity of the method for clinical implementation. Finally, the demonstrated stability of both extracted plasma analytes and unprocessed samples gives us a wide time window, which facilitates routine work in clinical practice.

## 6. Future Directions

The next step following the development and analytical validation of this UHPLC-UV/Vis method is its broader clinical implementation and clinical validation. Future studies should focus on evaluating the impact of routine application of this method on the optimization of beta-lactam therapy through therapeutic drug monitoring (TDM). By assessing clinical outcomes, dosing adjustments, and pharmacokinetic target attainment in real-world settings, it will be possible to determine the clinical utility and effectiveness of the method beyond the analytical level. Successful validation in a clinical context could strengthen the case for systematic TDM of beta-lactams and foster its adoption in other hospitals. This, in turn, may improve the rational use of beta-lactam antibiotics, enhance treatment efficacy, reduce toxicity, and contribute to combating antimicrobial resistance.

## Figures and Tables

**Table 1:** Chromatographic information (detector wavelength (λ) and retention time (t_R_)) and linearity details (calibration range, equation, coefficient of determination (R^2^), lower limit of quantitation (LLOQ), and precision at LLOQ (%MRE at LLOQ)).

Analyte	λ (nm)	t_R_ (min)	Calibration Range	Equation	R^2^	LLOQ	%MRE at LLOQ
FEP	260	1.2	2.5–50 mg/L	y = 0.54x + 0.62	0.9996	2.5 mg/L	6.1%
TOL	260	1.7	5–50 mg/L	y = 0.066x + 0.03	0.9978	5.0 mg/L	8.8%
CAZ	260	3.0	1–50 mg/L	y = 0.70x − 0.07	0.9993	1.0 mg/L	4.8%
MER	304	3.4	1–50 mg/L	y = 1.74x + 0.94	0.9995	1.0 mg/L	4.7%
AMP	210	4.0	1–50 mg/L	y = 1.19x + 0.41	0.9995	1.0 mg/L	5.1%
ETP	304	5.8	2.5–50 mg/L	y = 0.69x + 0.02	0.9987	2.5 mg/L	5.5%

**Table 2:** Accuracy and precision data of quality controls and dilution integrity.

Analyte	Within-Day Mean (mg/L) (%CV,%MRE)					Between-Day Mean (mg/L) (%CV,%MRE)				
	Quality Controls			Dilution Integrity		Quality Controls			Dilution Integrity	
	Low (5 mg/L)	Medium (20 mg/L)	High (50 mg/L)	ULOQ I (100 mg/L)	ULOQ II (150 mg/L)	Low (5 mg/L)	Medium (20 mg/L)	High (50 mg/L)	ULOQ I (100 mg/L)	ULOQ II (150 mg/L)
FEP	4.99 (0.1%, 6.5%)	17.74 (0.2%, 11.3%)	47.51 (0.1%, 4.8%)	95.17 (0.3%, 4.8%)	161.89 (0.3%, 7.9%)	5.50 (14.3%, 9.9%)	20.37 (7.4%, 1.9%)	49.64 (6.4%, 0.7%)	94 (1.1%, 5.7%)	165 (2.2%, 10.1%)
TOL	4.69 (2.2%, 6.1%)	20.38 (0.9%, 1.9%	53.5 (2.7%, 7.0%)	88.83 (0.1%, 11.1%)	157.50 (0.2%, 5.0%)	4.59 (2.8%, 8.3%)	20.24 (1.2%, 1.2%)	53.74 (6.9%, 7.5%)	85.04 (4.8%, 14.9%)	159.24 (1.5%, 6.1%)
CAZ	4.99 (0.1%, 0.1%)	18.95 (0.1%, 5.2%)	48.20 (2.7%, 3.6%)	95.17 (0.3%, 4.8%)	144.61 (0.1%, 3.6%)	5.01 (5%, 0.3%)	20.84 (6.7%, 4.2%)	51.93 (5.8%, 3.9%)	94.25 (1.1%, 5.7%)	151.53 (4.8%, 1.0%)
MER	5.23 (2.2%, 6.1%)	19.52 (0.2%, 2.4%)	50.00 (0.2%, 0.0%)	85.37 (0.2%, 14.6%)	161.41 (0.2%, 7.6%)	5.11 (2.6%, 2.2%)	19.30 (4.9%, 3.5%)	49.46 (8.3%, 1.1%)	85.20 (0.9%, 14.7%)	144.79 (12.1%, 3.5%)
AMP	5.16 (9.0%, 3.2%)	20.74 (2.5%, 3.7%)	51.15 (0.2%, 2.3%)	93.89 (0.7%, 6.1%)	161.11 (2.2%, 7.4%)	4.78 (11.6%, 4.5%)	20.78 (10.4%, 3.9%)	50.32 (5.8%, 0.6%)	93.92 (0.7%, 6.1%)	160.03 (1.8%, 6.7%)
ETP	5.33 (0.1%, 6.5%)	17.74 (0.2%, 11.1%)	47.61 (0.1%, 4.8%)	88.33 (0.5%, 11.6%)	150.43 (0.1%, 0.3%)	5.74 (9.1%, 14.8%)	19.46 (8.8%, 2.7%)	47.98 (8.4%, 4.0%)	85.3 (3.7%, 14.6%)	131.70 (8.8%, 10.6%)

**Table 3:** Stability test for beta-lactam antibiotics in water stored at −20 °C. Peak area: area under the chromatographic peak.

Antibiotic	Low 5 mg/L			High 50 mg/L		
	Peak Area from Fresh Solution	Peak Area From Solution Stored at −20 °C for 6 Months	Difference (%)	Peak Area from Fresh Solution	Peak Area from Solution Stored at −20 °C for 6 Months	Difference (%)
FEP	141,798.5	157,414.0	11.01 ± 0.13	1,370,465.0	1,526,423.5	11.37 ± 0.03
TOL	76,984.5	73,137.0	−4.99 ± 0.11	744,373.5	699,744.0	−5.99 ± 0.07
CAZ	236,617.0	209,564.5	−11.4 ± 0.03	2,282,023.0	1,951,511.0	−14.5 ± 0.01
MER	133,465.0	114,503.0	−14.2 ± 0.08	1,291,916.5	1,096,498.0	−14.8 ± 0.15
AMP	223,954.3	203,698.7	−9.0 ± 1.5	2,079,699.0	1,951,500.5	−6.2 ± 0.18
ETP	104,775.5	83,711.0	−20.1 ± 0.04	1,029,813.0	797,063.5	−22.6 ± 0.014
	**Medium 25 mg/L**			**Medium 25 mg/L**		
	**Peak area from fresh solution**	**Peak area from solution stored at −20 °C for 6 months**	**Difference (%)**	**Peak area from fresh solution**	**Peak area from solution stored at −20 °C for 9 months**	**Difference (%)**
IS	1157,708.5	1257,436.0	9.7 ± 0.24	1157,708.5	1172,750.0	1.3 ± 0.4

**Table 4:** Stability test for processed samples stored at −20 °C. Peak area: area under the chromatographic peak.

Antibiotic	Low 5 mg/L			Medium 20 mg/L			High 50 mg/L		
	Peak Area from Fresh Solution	Peak Area from Solution Stored at −20 °C for 1 month	Difference (%)	Peak Area from Fresh Solution	Peak Area from Solution Stored at −20 °C for 1 month	Difference (%)	Peak Area from Fresh Solution	Peak Area from Solution Stored at −20 °C for 1 month	Difference (%)
FEP	32,625.0	29,158.3	−10.62 ± 0.7	123,900.3	104,940.7	−15.30 ± 0.1	306,269.0	285,653.7	−6.7 ± 0.04
TOL	5070.0	5615.3	10.8 ± 2.2	24,996.0	23,176.0	−6.9 ± 0.1	45,603.0	40,493.3	−11.2 ± 0.35
CAZ	46,166.3	43,545.3	−5.68 ± 0.2	170,085.3	175,213.3	3.01 ± 0.04	417,308.0	400,512.3	−4.0 ± 0.05
MER	29,229.7	24,032.0	−17.78 ± 0.3	101,248.7	112,355.0	11.0 ± 0.2	254,201.3	250,520.3	−1.4 ± 0.06
AMP	62,630.3	62,674.0	0.054 ± 3.1	217,591.7	208,389.3	−4.2 ± 3.4	547,094.3	517,966.7	−5.2 ± 4.15
ETP	9758.7	10,005.3	2.55 ± 1.93	41,269.7	49,095.0	19.0 ± 0.04	107,943.0	90,978.7	−15.71 ± 0.09

## Data Availability

The original contributions presented in this study are included in the article/Supplementary Material. Further inquiries can be directed to the corresponding authors.

## Supplementary Materials

The following supporting information can be downloaded at https://www.mdpi.com/article/10.3390/antibiotics14060613/s1. Figure S1. Stability assay on different matrixes. (A) Whole blood (B) Plasma. Figure S2. Stability assay of analytes in extracted plasma in the autosample. (A) Processed samples from whole blood (B) Processed samples from plasma.

## References

[1] β-Lactams and β-Lactamase Inhibitors: An Overview–PubMed. https://pubmed.ncbi.nlm.nih.gov/27329032/.

[2] Verdier M.-C., Tribut O., Tattevin P., Le Tulzo Y., Michelet C., Bentué-Ferrer D. (2011). Simultaneous determination of 12 beta-lactam antibiotics in human plasma by high-performance liquid chromatography with UV detection: Application to therapeutic drug monitoring. Antimicrob. Agents Chemother..

[3] Abdul-Aziz M.H., Alffenaar J.-W.C., Bassetti M., Bracht H., Dimopoulos G., Marriott D., Neely M.N., Paiva J.-A., Pea F., Sjovall F. (2020). Antimicrobial therapeutic drug monitoring in critically ill adult patients: A Position Paper. Intensive Care Med..

[4] Pai Mangalore R., Ashok A., Lee S.J., Romero L., Peel T.N., Udy A.A., Peleg A.Y. (2022). Beta-Lactam Antibiotic Therapeutic Drug Monitoring in Critically Ill Patients: A Systematic Review and Meta-Analysis. Clin. Infect. Dis..

[5] Veiga R.P., Paiva J.-A. (2018). Pharmacokinetics-pharmacodynamics issues relevant for the clinical use of beta-lactam antibiotics in critically ill patients. Crit. Care.

[6] Roberts J.A., Ulldemolins M., Roberts M.S., McWhinney B., Ungerer J., Paterson D.L., Lipman J. (2010). Therapeutic drug monitoring of beta-lactams in critically ill patients: Proof of concept. Int. J. Antimicrob. Agents.

[7] Novy E., Martinière H., Roger C. (2023). The Current Status and Future Perspectives of Beta-Lactam Therapeutic Drug Monitoring in Critically Ill Patients. Antibiotics.

[8] Briscoe S.E., McWhinney B.C., Lipman J., Roberts J.A., Ungerer J.P.J. (2012). A method for determining the free (unbound) concentration of ten beta-lactam antibiotics in human plasma using high performance liquid chromatography with ultraviolet detection. J. Chromatogr. B Analyt. Technol. Biomed. Life. Sci..

[9] McWhinney B.C., Wallis S.C., Hillister T., Roberts J.A., Lipman J., Ungerer J.P.J. (2010). Analysis of 12 beta-lactam antibiotics in human plasma by HPLC with ultraviolet detection. J. Chromatogr. B Analyt. Technol. Biomed. Life. Sci..

[10] Bricheux A., Lenggenhager L., Hughes S., Karmime A., Lescuyer P., Huttner A. (2019). Therapeutic drug monitoring of imipenem and the incidence of toxicity and failure in hospitalized patients: A retrospective cohort study. Clin. Microbiol. Infect..

[11] Ikeda K., Morikawa N., Kuribayashi M., Ikawa K., Nomura K., Taniwaki M. (2007). Real-time therapeutic drug monitoring of cefozopran in plasma using high-performance liquid chromatography with ultraviolet detection. J. Pharm. Biomed. Anal..

[12] Eldougdoug M.W., Youssef D.M., El-Shal A.S., Sharaf Y.A., Raparla S., Jasti B.R., Elnahas H.M. (2023). Evaluation of ceftriaxone pharmacokinetics in hospitalized Egyptian pediatric patients. Eur. J. Pediatr..

[13] Denooz R., Charlier C. (2008). Simultaneous determination of five beta-lactam antibiotics (cefepim, ceftazidim, cefuroxim, meropenem and piperacillin) in human plasma by high-performance liquid chromatography with ultraviolet detection. J. Chromatogr. B Analyt. Technol. Biomed. Life. Sci..

[14] Zaragoza R., Vidal-Cortés P., Aguilar G., Borges M., Diaz E., Ferrer R., Maseda E., Nieto M., Nuvials F.X., Ramirez P. (2020). Update of the treatment of nosocomial pneumonia in the ICU. Crit. Care.

[15] D’Cunha R., Bach T., Young B.A., Li P., Nalbant D., Zhang J., Winokur P., An G. (2018). Quantification of Cefepime, Meropenem, Piperacillin, and Tazobactam in Human Plasma Using a Sensitive and Robust Liquid Chromatography-Tandem Mass Spectrometry Method, Part 1: Assay Development and Validation. Antimicrob. Agents Chemother..

[16] Barreto E.F., Chitre P.N., Pine K.H., Shepel K.K., Rule A.D., Alshaer M.H., Abdul Aziz M.H., Roberts J.A., Scheetz M.H., Ausman S.E. (2023). Why is the Implementation of Beta-Lactam Therapeutic Drug Monitoring for the Critically Ill Falling Short? A Multicenter Mixed-Methods Study. Ther. Drug Monit..

[17] Venugopalan V., Hamza M., Santevecchi B., DeSear K., Cherabuddi K., Peloquin C.A., Alshaer M.H. (2022). Implementation of a β-lactam therapeutic drug monitoring program: Experience from a large academic medical center. Am. J. Health Syst. Pharm.

[18] Antibiotics in Serum/Plasma–HPLC. https://chromsystems.com/en/antibiotics-in-serum-plasma-hplc-61000.html.

[19] Guilhaumou R., Benaboud S., Bennis Y., Dahyot-Fizelier C., Dailly E., Gandia P., Goutelle S., Lefeuvre S., Mongardon N., Roger C. (2019). Optimization of the treatment with beta-lactam antibiotics in critically ill patients-guidelines from the French Society of Pharmacology and Therapeutics (Société Française de Pharmacologie et Thérapeutique-SFPT) and the French Society of Anaesthesia and Intensive Care Medicine (Société Française d’Anesthésie et Réanimation-SFAR). Crit. Care.

[20] Shipkova M., López O.M., Picard N., Noceti O., Sommerer C., Christians U., Wieland E. (2016). Analytical Aspects of the Implementation of Biomarkers in Clinical Transplantation. Ther. Drug Monit..

[21] Adaway J.E., Keevil B.G., Owen L.J. (2015). Liquid chromatography tandem mass spectrometry in the clinical laboratory. Ann. Clin. Biochem..

[22] Parker S.L., Pandey S., Sime F.B., Stuart J., Lipman J., Roberts J.A., Wallis S.C. (2021). A validated LC-MS/MS method for the simultaneous quantification of the novel combination antibiotic, ceftolozane-tazobactam, in plasma (total and unbound), CSF, urine and renal replacement therapy effluent: Application to pilot pharmacokinetic studies. Clin. Chem. Lab. Med..

[23] Mula J., Chiara F., Manca A., Palermiti A., Maiese D., Cusato J., Simiele M., De Rosa F.G., Di Perri G., De Nicolò A. (2023). Analytical validation of a novel UHPLC-MS/MS method for 19 antibiotics quantification in plasma: Implementation in a LC-MS/MS Kit. Biomed. Pharmacother..

[24] Rigo-Bonnin R., Gomez-Junyent J., García-Tejada L., Benavent E., Soldevila L., Tubau F., Murillo O. (2019). Measurement of ceftolozane and tazobactam concentrations in plasma by UHPLC-MS/MS. Clinical application in the management of difficult-to-treat osteoarticular infections. Clin. Chim. Acta.

[25] Ezquer-Garin C., Ferriols-Lisart R., Alós-Almiñana M., Aguilar-Aguilar G., Belda-Nacher J.F., Carbonell J.-A. (2018). Validated HPLC-UV detection method for the simultaneous determination of ceftolozane and tazobactam in human plasma. Bioanalysis.

[26] Toullec L., Dupouey J., Vigne C., Marsot A., Allanioux L., Blin O., Leone M., Guilhaumou R. (2017). Analytical Interference During Cefepime Therapeutic Drug Monitoring in Intensive Care Patient: About a Case Report. Therapies.

[27] Bioanalytical Method Validation–Scientific Guideline European Medicines Agency (EMA). https://www.ema.europa.eu/en/bioanalytical-method-validation-scientific-guideline.

[28] Center for Drug Evaluation and Research, Center for Veterinary Medicine Bioanalytical Method Validation Guidance for Industry. https://www.fda.gov/regulatory-information/search-fda-guidance-documents/bioanalytical-method-validation-guidance-industry.

[29] Rigo-Bonnin R., Ribera A., Arbiol-Roca A., Cobo-Sacristán S., Padullés A., Murillo Ò., Shaw E., Granada R., Pérez-Fernández X.L., Tubau F. (2017). Development and validation of a measurement procedure based on ultra-high performance liquid chromatography-tandem mass spectrometry for simultaneous measurement of β-lactam antibiotic concentration in human plasma. Clin. Chim. Acta.

[30] Carlier M., Stove V., De Waele J.J., Verstraete A.G. (2015). Ultrafast quantification of β-lactam antibiotics in human plasma using UPLC–MS/MS. J. Chromatogr. B.

[31] Colin P., De Bock L., T’jollyn H., Boussery K., Van Bocxlaer J. (2013). Development and validation of a fast and uniform approach to quantify β-lactam antibiotics in human plasma by solid phase extraction-liquid chromatography–electrospray-tandem mass spectrometry. Talanta.

[32] Ohmori T., Suzuki A., Niwa T., Ushikoshi H., Shirai K., Yoshida S., Ogura S., Itoh Y. (2011). Simultaneous determination of eight β-lactam antibiotics in human serum by liquid chromatography–tandem mass spectrometry. J. Chromatogr. B.

[33] Cohen-Wolkowiez M., White N.R., Bridges A., Benjamin D.K., Kashuba A.D.M. (2011). Development of a liquid chromatography–tandem mass spectrometry assay of six antimicrobials in plasma for pharmacokinetic studies in premature infants. J. Chromatogr. B.

[34] Rhodes A., Evans L.E., Alhazzani W., Levy M.M., Antonelli M., Ferrer R., Kumar A., Sevransky J.E., Sprung C.L., Nunnally M.E. (2017). Surviving Sepsis Campaign: International Guidelines for Management of Sepsis and Septic Shock: 2016. Intensive Care Med..

[35] Stašek J., Keller F., Kočí V., Klučka J., Klabusayová E., Wiewiorka O., Strašilová Z., Beňovská M., Škardová M., Maláska J. (2023). Update on Therapeutic Drug Monitoring of Beta-Lactam Antibiotics in Critically Ill Patients-A Narrative Review. Antibiotics.

[36] European Committee on Antimicrobial Susceptibility Testing (EUCAST) Antimicrobial Wild Type Distributions of Microorganisms. https://mic.eucast.org/search/.

[37] Bahmany S., Ewoldt T.M.J., Abdulla A., Koch B.C.P. (2023). Stability of 10 Beta-Lactam Antibiotics in Human Plasma at Different Storage Conditions. Ther. Drug Monit..

[38] Gijsen M., Filtjens B., Annaert P., Armoudjian Y., Debaveye Y., Wauters J., Slaets P., Spriet I. (2021). Meropenem Stability in Human Plasma at -20 °C: Detailed Assessment of Degradation. Antibiotics.

[39] Preanalytical Stability of 13 Antibiotics in Biological Samples: A Crucial Factor for Therapeutic Drug Monitoring. https://www.mdpi.com/2079-6382/13/7/675.

[40] Roberts J.A., Paul S.K., Akova M., Bassetti M., De Waele J.J., Dimopoulos G., Kaukonen K.-M., Koulenti D., Martin C., Montravers P. (2014). DALI: Defining antibiotic levels in intensive care unit patients: Are current β-lactam antibiotic doses sufficient for critically ill patients?. Clin. Infect. Dis..

[41] Sime F.B., Roberts M.S., Peake S.L., Lipman J., Roberts J.A. (2012). Does Beta-lactam Pharmacokinetic Variability in Critically Ill Patients Justify Therapeutic Drug Monitoring? A Systematic Review. Ann. Intensive Care.

[42] Gonçalves-Pereira J., Póvoa P. (2011). Antibiotics in critically ill patients: A systematic review of the pharmacokinetics of β-lactams. Crit. Care.

[43] Boidin C., Moshiri P., Dahyot-Fizelier C., Goutelle S., Lefeuvre S. (2020). Pharmacokinetic variability of beta-lactams in critically ill patients: A narrative review. Anaesth. Crit. Care Pain. Med..

[44] Fratoni A.J., Nicolau D.P., Kuti J.L. (2021). A guide to therapeutic drug monitoring of β-lactam antibiotics. Pharmacother. J. Hum. Pharmacol. Drug Ther..

[45] Schießer S., Hitzenbichler F., Kees M.G., Kratzer A., Lubnow M., Salzberger B., Kees F., Dorn C. (2021). Measurement of Free Plasma Concentrations of Beta-Lactam Antibiotics: An Applicability Study in Intensive Care Unit Patients. Ther. Drug Monit..

[46] Ulldemolins M., Roberts J.A., Rello J., Paterson D.L., Lipman J. (2011). The Effects of Hypoalbuminaemia on Optimizing Antibacterial Dosing in Critically Ill Patients. Clin. Pharmacokinet..

[47] European Committee on Antimicrobial Susceptibility Testing (EUCAST) Public Consultations. https://www.eucast.org/publications-and-documents/consultations.

