# Supplementary material for: Development of a UHPLC-UV/Vis Method for Simultaneously Determining Six Beta-Lactam Antibiotics in Plasma: A Tool for the Clinical Implementation of Therapeutic Monitoring of Beta-Lactams
Source: Antibiotics (Basel). 2025 Jun 17;14(6):613. doi: 10.3390/antibiotics14060613 (PMC12189362; doi:10.3390/antibiotics14060613)

SUPPLEMENTARY MATERIAL

Figure S1: Stability assay on different matrixes. A) Whole blood B) Plasma

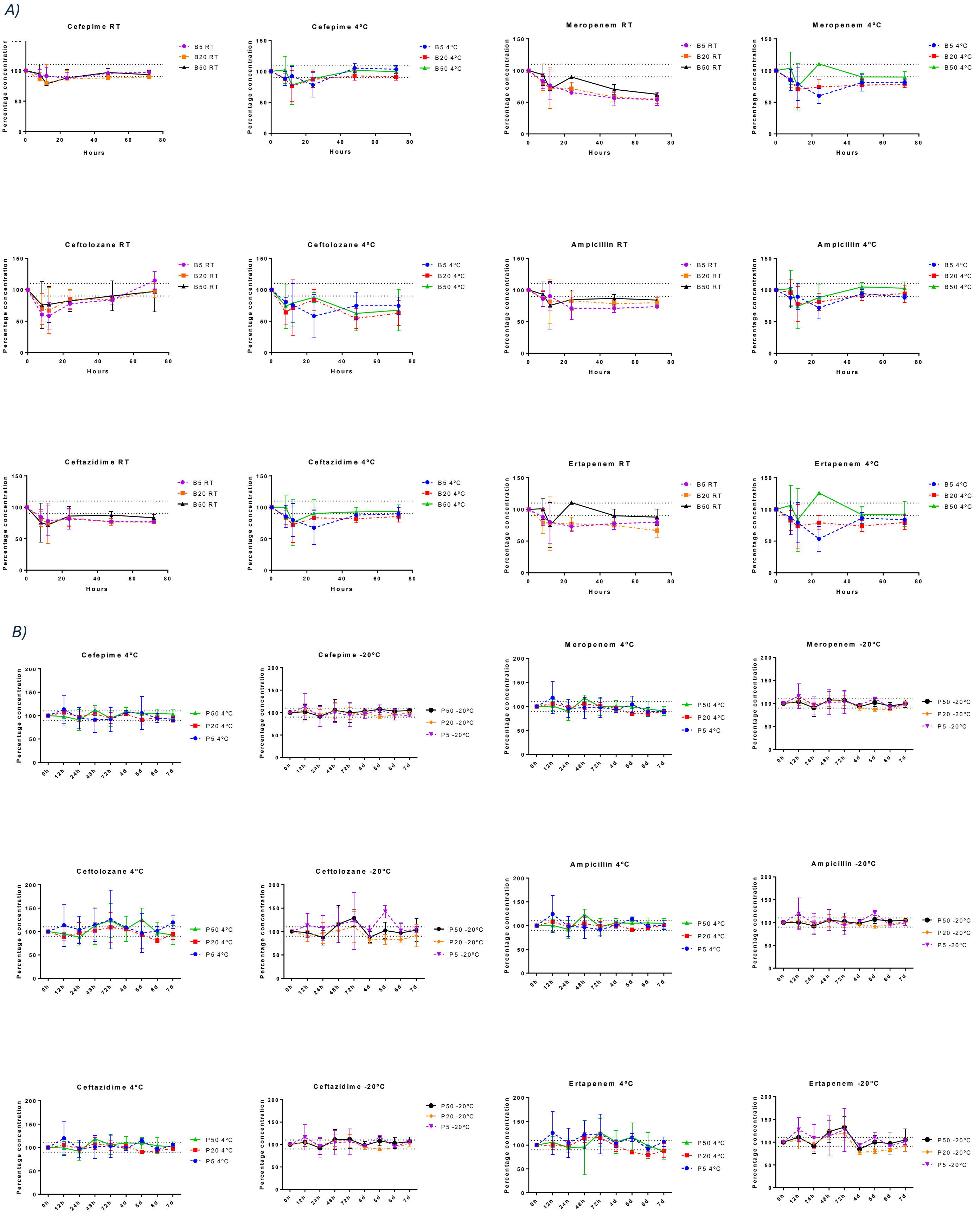

Figure S2: Stability assay of analytes in extracted plasma in the autosample. A) Processed samples from whole blood B) Processed samples from plasma.

A)

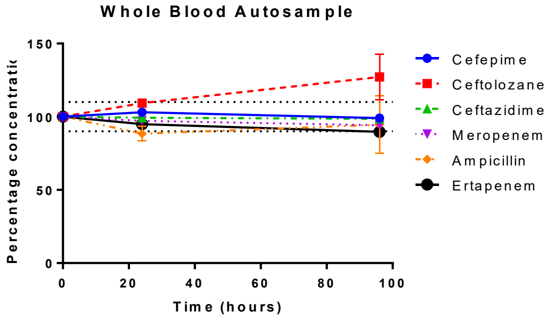

B)

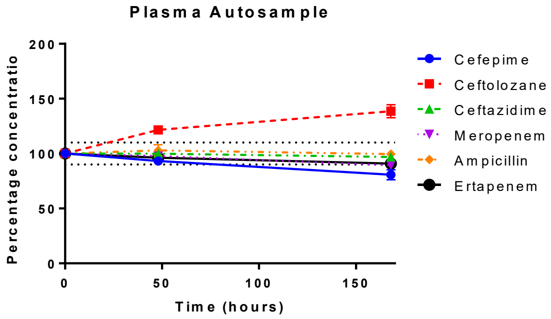

Supplement: Supplementary file 1 [file antibiotics-14-00613-s001.zip › antibiotics-3645193-supplementary.pdf]
